# The Cost-Effectiveness of Tuberculosis Preventive Therapy for HIV-Infected Individuals in Southern India: A Trial-Based Analysis

**DOI:** 10.1371/journal.pone.0036001

**Published:** 2012-04-30

**Authors:** Mai T. Pho, Soumya Swaminathan, Nagalingeswaran Kumarasamy, Elena Losina, C. Ponnuraja, Lauren M. Uhler, Callie A. Scott, Kenneth H. Mayer, Kenneth A. Freedberg, Rochelle P. Walensky

**Affiliations:** 1 Section of Hospital Medicine, Department of Medicine, University of Chicago Medical Center, Chicago, Illinois, United States of America; 2 National Institute for Research in Tuberculosis, Chennai, India; 3 Y. R. Gaitonde Centre for AIDS Research and Education, VHS, Chennai, India; 4 Division of General Medicine; 5 Division of Infectious Diseases, Massachusetts General Hospital, Harvard Medical School, Boston, Massachusetts, United States of America; 6 Division of Infectious Diseases, Brigham and Women’s Hospital, Boston, Massachusetts, United States of America; 7 Department of Orthopedic Surgery, Brigham and Women’s Hospital, Boston, Massachusetts, United States of America; 8 Harvard University Center for AIDS Research, Harvard Medical School, Boston, Massachusetts, United States of America; 9 Department of Biostatistics, Boston University School of Public Health, Boston Massachusetts, United States of America; 10 Department of Epidemiology, Boston University School of Public Health, Boston Massachusetts, United States of America; 11 Department of Health Policy and Management, Harvard School of Public Health, Boston, Massachusetts, United States of America; 12 Miriam Hospital, Brown University, Providence, Rhode Island, United States of America; McGill University, Canada

## Abstract

**Background:**

Regimens for isoniazid-based preventive therapy (IPT) for tuberculosis (TB) in HIV-infected individuals have not been widely adopted given concerns regarding efficacy, adherence and drug resistance. Further, the cost-effectiveness of IPT has not been studied in India.

**Methods:**

We used an HIV/TB model to project TB incidence, life expectancy, cost and incremental cost-effectiveness of six months of isoniazid plus ethambutol (6EH), thirty-six months of isoniazid (36H) and no IPT for HIV-infected patients in India. Model input parameters included a median CD4 count of 324 cells/mm^3^, and a rate ratio of developing TB of 0.35 for 6EH and 0.22 for 36H at three years as compared to no IPT. Results of 6EH and 36H were also compared to six months of isoniazid (6H), three months of isoniazid plus rifampin (3RH) and three months of isoniazid plus rifapentine (3RPTH).

**Results:**

Projected TB incidence decreased in the 6EH and 36H regimens by 51% and 62% respectively at three-year follow-up compared to no IPT. Without IPT, projected life expectancy was 136.1 months at a lifetime per person cost of $5,630. 6EH increased life expectancy by 0.8 months at an additional per person cost of $100 (incremental cost-effectiveness ratio (ICER) of $1,490/year of life saved (YLS)). 36H further increased life expectancy by 0.2 months with an additional per person cost of $55 (ICER of $3,120/YLS). The projected clinical impact of 6EH was comparable to 6H and 3RH; however when compared to these other options, 6EH was no longer cost-effective given the high cost of ethambutol. Results were sensitive to baseline CD4 count and adherence.

**Conclusions:**

Three, six and thirty-six-month regimens of isoniazid-based therapy are effective in preventing TB. Three months of isoniazid plus rifampin and six-months of isoniazid are similarly cost-effective in India, and should be considered part of HIV care.

## Introduction

The HIV and tuberculosis epidemics represent a major public health challenge in India [1], [2]. Among the estimated 2.4 million people living with HIV/AIDS in India, the incidence of active TB has been reported as high as 6.90 cases/100 person-years (PY) [3], [4]. This is driven in part by reactivation disease in the estimated 40% of HIV-infected persons latently infected with TB [5].

Given the challenge of controlling these epidemics, major stakeholders convened at the World Health Organization and identified intensified TB case-finding, infection control, and isoniazid-based preventive therapy (IPT) as crucial measures in reducing the impact of TB on people living with HIV [6]. In India’s governmental response to the epidemics, the Central TB Division (CTD) and National AIDS Control Organisation (NACO) initiated an “intensified TB-HIV package" of services including routine HIV testing for TB patients and enhanced TB monitoring [7]. While IPT was not recommended, the cost-effectiveness of IPT was listed as a priority research area [7]. Our objective was to examine the cost-effectiveness of IPT for HIV-infected patients in India.

## Methods

### Analytic Overview

We incorporated data from the National Institute for Research in Tuberculosis clinical trial entitled “Preventive Therapy for TB in HIV-Infected Patients" in India into a previously published model of HIV/TB disease to project the long-term clinical and economic impact of alternative strategies of IPT [8], [9], [10], [11]. The internal validity of the model was assessed by comparing projected incidence of active TB in the IPT strategies at three years to outcomes of the trial [9]. Model outcomes included the incidence of active TB and cost per person at three and ten years (2009 USD), projected life expectancy, lifetime per person costs and cost-effectiveness measured in incremental cost per year of life saved (YLS). Life expectancy and costs were discounted at 3% per year [12]. As suggested by the WHO Commission on Macroeconomics and Health, an IPT strategy was considered to be “cost-effective" if the incremental cost-effectiveness ratio was less than three times the Gross Domestic Product (GDP) *per capita* (for India, GDP *per capita* was $980, 3x GDP *per capita* was $2,940 in 2009 USD [13], [14]Sensitivity analyses were performed to test the stability of model outputs as parameters were varied.

### Model Structure

We utilized the Cost-Effectiveness of Preventing AIDS Complications (CEPAC)-International model, a computer-based, state transition model of HIV and TB combined with data on epidemiology and treatment in India. Briefly, individually-simulated, antiretroviral therapy (ART)-naïve patients without past history of TB entered the model and moved on a monthly cycle through health states characterized by chronic HIV infection, active TB, opportunistic diseases, drug toxicity and death. Transition probabilities between health states were determined by CD4 count, HIV RNA level, response to ART, and prior history of opportunistic infection (OI) [15], [16]. Incidence of active TB included reactivation of latent disease and exogenous infection; incidence decreased with use of ART [17]. According to NACO guidelines, co-trimoxazole was initiated at a CD4 threshold of <200 cells/mm^3^ [18]. Non-nucleoside reverse transcriptase inhibitor-based first-line ART was initiated at a CD4 threshold <250 cells/mm^3^ or <350 cells/mm^3^ in the setting of a stage III AIDS-related OI [19]. Patients who failed first-line ART were offered second-line protease inhibitor-containing therapy [19].

### Simulated Strategies

Model strategies were based on the intervention arms of an open-label, randomized trial completed by the National Institute for Research in Tuberculosis in Chennai between 2001–2008 comparing the efficacy of two regimens of IPT, a six-month course of isoniazid plus ethambutol (6EH) and thirty-six month course of isoniazid (36H) [9]. The primary outcome of the trial was TB incidence at three-year follow-up. Secondary outcomes were all cause mortality and adverse events. As there was no placebo arm in the trial, a “no IPT" strategy was simulated based on previously published natural history data from the National Institute for Research in Tuberculosis [3]. To remain conservative, IPT was assumed to have no benefit after treatment discontinuation, against drug-resistant TB, and for any patient who did not complete the full six-or thirty-six month regimens. We assumed that the risk of re-infection of TB after IPT discontinuation returned to baseline TB incidence.

To contextualize the findings of 6EH and 36H within current WHO recommendations for IPT of six months of isoniazid alone, as well as recent trial results evaluating the use of shorter, rifamycin-containing regimens for TB prevention in HIV patients, an additional analysis was performed comparing the incremental cost-effectiveness of 6EH and 36H with six months of isoniazid (300 mg) daily (6H), three months of isoniazid (900 mg) plus rifampin (600 mg) twice weekly (3RH), and three months of isoniazid (900 mg) plus rifapentine (900 mg) weekly (3RPTH). All IPT strategies included pyridoxine daily. These IPT regimens were selected based on WHO guidelines for IPT in resource-limited settings, as well as recent efficacy data published in Botswana and South Africa in HIV-infected patients [20], [21], [22].

### Input Data

#### Cohort Characteristics

Baseline cohort characteristics were taken from the National Institute for Research in Tuberculosis trial (Table 1) [9]. The mean age was 30, 63% of patients were female, and the median CD4 count was 324 cells/mm^3^. Twenty-seven percent of patients met criteria and were initiated on ART.

**Table 1:** Baseline cohort characteristics, TB and HIV natural history, HIV treatment parameters.

Parameter	Base Case Value	Range for Sensitivity Analysis	References
**Cohort characteristics**			
Mean age (years), SD	30, 7		[9]
Gender (% female)	63		[9]
Median CD4 (cells/mm^3^), IQR	324, 200–506	100–500	[9]
% receiving ART	27		[9]
**TB Natural History**			
TB Incidence/100 PY	6.90	5.0–10.0	[3], [9], [24]
Drug resistant TB			
All isoniazid-resistant TB (%)	16%	8%–64%	[23]
MDR-TB (%)	6%	3%–24%	[23]
Probability of death with TB, range by CD4 strata^a^			
Non-MDR TB	0–0.50	0–0.74	[27], [28], [56]
MDR-TB	0–0.56	0–0.84	[27], [28], [56]
**Natural history of HIV**			
Monthly CD4 decline (cells/mm^3^) by viral load			
>30,000 copies/ml	6.4	3.2–12.8	[55]
10,001–30,000 copies/ml	5.4	2.7–10.8	[55]
3,001–10,000 copies/ml	4.6	2.3–9.2	[55]
501–3,000 copies/ml	3.7	1.85–7.4	[55]
< = 500 copies/ml	3.0	1.5–6.0	[55]
**Efficacy of ART for first and second line ART**			
HIV RNA suppression at 48 weeks (%)	73	66–80	[32]
CD4 cell increase at 24 weeks (cells/mm^3^)	148	133–163	[31]

**SD**: Standard deviation; **IQR**: Interquartile range; **TB**: Tuberculosis**; PY**: Person-Years; **MDR**: Multidrug-resistant; **ART**: Antiretroviral therapy.

a Probability of death within 6 months after active TB.

#### HIV and TB Natural History

In the absence of ART, patients experienced a decline in CD4 count of 3.0–6.4 cells/mm^3^ per month as determined by their HIV RNA level [16]. The base case incidence of active TB among HIV-infected patients without IPT (6·.90/100 PY) was obtained from previously published data [3]. The prevalence of isoniazid-resistant TB was 16%; 6% of patients developed multidrug-resistant (MDR-TB), defined by resistance to at least isoniazid and rifampin [23]. The incidences of other OIs, including cryptoccocus, esophageal candidiasis, *Pneumocystis jiroveci* pneumonia, toxoplasmosis, Kaposi’s sarcoma, cytomegalovirus infection, and progressive multifocal encephalopathy were obtained from the Y. R. Gaitonde Centre for AIDS Research and Education (YRG CARE) cohort in Chennai [24], [25]. In the absence of data from India, primary data from Côte d’Ivoire were used for the mortality associated with AIDS and OIs, and from the US for baseline CD4 decline [16], [26], [27], [28]. These parameters were tested in sensitivity analysis.

#### The Efficacy of IPT

The efficacies of the IPT strategies in the model were based on the TB incidence at three-year follow-up observed in the intent-to-treat analysis of the 6EH and 36H arms of the National Institute for Research in Tuberculosis trial. A rate ratio was calculated by comparing these incidences to the baseline TB incidence of 6.90 cases/100 PY as assumed for the no IPT strategy [9]. The incidence of active TB in the 6EH arm was 2.44 cases/100 PY (95% CI 1.42–3.46), resulting in an incidence rate ratio for the 6EH strategy of 0.35 compared to no IPT [9]. The incidence of active TB in the 36H arm was 1.55 cases/100 PY (95% CI 0.73–2.36), resulting in an incidence rate ratio for the 36H strategy of 0.22 compared to no IPT [9]. Projections were based on the assumption that the difference between the point estimates of efficacy for 6EH and 36H were significant. Because this difference did not reach significance in the trial, likely due to a low event rate and study power, sensitivity analyses were performed to examine the optimal scenarios in which the 36H strategy was cost-effective compared to the 6EH strategy.

In the absence of data from India, and the modeled trial specifically, the efficacy of alternative IPT strategies in HIV-infected patients was estimated from trials performed in HIV-infected persons in sub-Saharan Africa. The relative incidence of TB with 6H compared to continuous isoniazid was obtained using trial data from Botswana [21]. This study was selected given a similar randomized trial structure as that performed in India in the post-ART era. A rate ratio comparing this incidence with the baseline incidence of TB from sub-Saharan Africa, adjusted by the availability of ART was calculated to be 0.39. The efficacy of 3RH and 3RPTH was similarly calculated using data from South Africa and both found to be 0.32 [20], [29].

#### Active TB Treatment

Active TB in patients who had not received IPT was treated with a Category I regimen, consisting of initiation with isoniazid, rifampin, pyrazinamide, and ethambutol thrice weekly for two months, followed by isoniazid and rifampin for four months as defined by the Revised National TB Control Programme (RNTCP) of India [7]. In patients who received IPT, cases of active TB were considered treatment failures and were treated with a Category II regimen, consisting of isoniazid, rifampin, pyrazinamide, ethambutol thrice-weekly for eight months, and the addition of an injectable aminoglycoside thrice-weekly for the first two months [7]. Of the patients with isoniazid-resistant, non MDR-TB, 18% were presumed to fail Category I treatment and required Category II treatment [30]. Patients with MDR-TB sequentially failed Category I and Category II treatment, and were subsequently treated with an aminoglycoside, a quinolone, ethambutol, ethionamide and pyrazinamide for twenty-four months [7].

#### HIV Treatment

First-line ART consisted of stavudine, lamivudine, and nevirapine as recommended by NACO [19]. ART efficacy was based on data from the Therapeutics Research, Education, and AIDS Training in Asia (TREAT ASIA), and the Antiretroviral Therapy in Low-Income Countries (ART-LINC) cohorts, which reported 48-week virologic suppression of 73% and mean CD4 count increase of 148 cells/mm^3^ at 24 weeks [31], [32]. Second-line therapy consisted of tenofovir, lamivudine, and lopinovir/ritonavir [19]. In the absence of efficacy data for protease inhibitor-based regimens in India, the efficacy of second-line therapy was assumed to be similar to first line-therapy. This assumption was tested in sensitivity analysis.

#### Toxicity

The likelihood of toxicity associated with 6EH and 36H was obtained from the National Institute for Research in Tuberculosis trial. Minor toxicity, defined as rash, elevated aminotransferases, and mild neuropathy, did not change duration of treatment or treatment efficacy, and occurred with a probability of 0.03 in the 6EH and 36H strategies [9]. Major toxicity, defined as severe hepatitis or severe neuropathy leading to permanent discontinuation of treatment occurred in two patients in the 36H arm. As treatment was not permanently discontinued in any patients in the 6EH arm, we calculated a pooled probability of major toxicity of 0.0029 for both strategies [9]. There were no episodes of fatal hepatitis in either the 6EH or 36H strategies. The toxicities of alternate strategies of IPT were obtained from published data (Table 2). Since the likelihood of minor toxicity of 6H was not reported in the Botswana trial, we assumed it to be the same as 6EH. Major toxicity leading to discontinuation of the drug occurred in 1.4% of patients, with fatal hepatitis occurring in 0.1% of patients [21]. Drug discontinuation due to major toxicity or pregnancy in the 3RH and 3RPTH strategies occurred in 3.8% and 1.8% of patients, respectively [20].

**Table 2:** Efficacy and toxicity of IPT.

Parameter	Base Case Value	Range for Sensitivity Analysis	Ref.
**Efficacy of IPT, RR** a		95% CI	
6EH	0.35	0.21–0.5	[9]
36H	0.22	0.11–0.34	[9]
6H	0.39	0.20–0.60	[21]
3RH	0.32	0.16–0.48	[20]
3RPTH	0.32	0.16–0.48	[20]
**Adherence**			
Regimen completion IPT (%)	100	40–100	[9]
**Toxicity**			
Probability of IPT associated minor toxicity			
6EH	0.03	0–0.20	[9]
36H	0.03	0–0.20	[9]
6H	0.03	0–0.20	Assumed
3RH	0.07	0–0.20	[20]
3RPTH	0.06	0–0.20	[20]
Probability of IPT associated major toxicity^b^			
6EH	0.0029	0–0.20	[9]
36H	0.0029	0–0.20	[9]
6H	0.0140	0–0.20	[50]
3RH	0.0380	0–0.20	[20]
3RPTH	0.0180	0–0.20	[20]
Probability of fatal toxicity	0–0.001	0–0.20	[9]

**IPT**: Isoniazid-based Preventive Therapy; **RR**: Rate Ratio; **6EH**: Six-month regimen of isoniazid plus ethambutol; **36H**: Three-year regimen of isoniazid; **3RH**: Three-month regimen of isoniazid plus rifampin; **3RPTH**: Three-month regimen of isoniazid plus rifapentine.

a Efficacy defined by rate ratio of TB incidence in the 6EH and 36H IPT regimens compared to no IPT at three year follow-up.

b Within first month of treatment.

#### Resource Utilization and Cost

The costs of IPT included medication costs, the costs of pre-screening for active TB (symptom screen and exam as recommended by the WHO), quarterly clinic visits, and liver function tests every six months (Table 3) [33], [34], [35]. Costs for Category I, Category II, and MDR-TB treatment included previously published costs of acute disease, medication costs, and directly observed therapy (DOT) [8], [34], [35]. Costs of ART were obtained from NACO [36]. Resource utilization including mean outpatient and inpatient days for routine HIV care, laboratory monitoring including CD4 count, treatment of opportunistic infection, and toxicity were derived from YRG CARE [8], [33], [37].

**Table 3:** Selected cost parameters.

Parameter	Base Case Value	Range for Sensitivity Analysis	Ref.
Costs (2009 USD)			
Chest radiograph	1	0.30–3	[35]
TB sputum stain and culture	17	6–50	[33], [35]
Liver function test	9	3–27	[33]
DOT visit	1	0.30–3	[35]
IPT Regimens^a^			
Total 6EH course	40	19–133	[33], [34], [35]
Total 36H course	90	47–363	[33], [34], [35]
Total 6H course	20		[33], [34], [35]
Total 3RH course	25		[33], [34], [35]
Total 3RPTH course	180		[33], [34], [35], [57]
Active TB treatment^b^			
Drug-sensitive TB^c^	50	41–300	[8], [34], [35]
INH-resistant, non-MDR TB^d^	140	113–906	[8], [34], [35]
MDR-TB^e^	2,630	2,105–16,842	[8], [34], [35]
First-line ART (NNRTI-based), monthly	9	8–10	[36]
Second-line ART (PI-based), monthly	55	50–60	[36]

**USD**: US Dollars; **TB**: Tuberculosis; **DOT**: Directly Observed Therapy; **6EH**: Six-month regimen of isoniazid plus ethambutol; **36H**: Three-year regimen of isoniazid; **3RH**: Three-month regimen of isoniazid plus rifampin; **3RPTH**: Three-month regimen of isoniazid plus rifapentine; MDR: multidrug-resistant; **ART**: Antiretroviral Therapy; **NNRTI**: Non-nucleoside reverse transcriptase inhibitor; **PI**: Protease inhibitor; **3RPTH**: Three-month regimen of isoniazid plus rifapentine.

a Includes clinic visits, TB symptom screening quarterly, liver function tests every six months, and medications.

b Includes costs of acute presentation of TB (including inpatient and outpatient visits), plus TB treatment plus DOT.

c Category I (two-months intensive phase with isoniazid, rifampin, pyrazinamide, and ethambutol thrice-weekly, then four-months continuation phase with isoniazid and rifampin thrice-weekly) and DOT.

d Category I failure, followed by Category II treatment (three-months intensive phase with isoniazid, rifampin, pyrazinamide, and ethambutol thrice-weekly, with first two months including streptomycin thrice-weekly, then five-month continuation phase with isoniazid, rifampin and ethambutol thrice-weekly) and DOT.

e Category I and II failure, followed by MDRTB treatment (six-month intensive phase with streptomycin, ofloxacin, ethambutol, ethionamide, and pyrazinamide daily, followed by an eighteen-month of continuation phase with ofloxacin, ethambutol, ethionamide, and pyrazinamide daily) and DOT.

### Sensitivity Analysis

Efficacies of 6EH and 36H regimens were varied within the bounds of the 95% confidence interval obtained from the trial analysis [9]. The probability of IPT-related toxicity and associated probability of death from fatal hepatitis were varied in each regimen independently from 0–20% based on the literature [38]. Adherence to IPT was examined by varying the percent of the cohort achieving regimen completion. An ART starting criterio n≤350 cells/mm^3^ was examined given new WHO guidelines for ART initiation [39]. The costs of IPT and active TB treatment were varied to reflect estimates in the literature, as well as inclusion of intensified case-finding using AFB smear, culture and chest radiograph [40], [41], [42], [43]. The baseline incidence of TB, prevalence of drug-resistant TB, mean CD4 count at model entry, rates of CD4 decline, and CD4-specific mortality were also varied to examine the effect of population heterogeneity. Multi-way analyses of parameters were examined to assess their policy impact.

## Results

### Base Case Analysis and Model Validation

At three-year follow-up, model projections for TB incidences in the no IPT, 6EH, and 36H strategies were 4.54, 2.23, and 1.74 cases/100 PY (Table 4). The estimates lay within the 95% confidence interval of the trial results [9]. Rates for all strategies trended towards convergence at ten years. At three-year follow-up, per-person costs were $680, $720, and $770 in the no IPT, 6EH and 36H strategies, and at ten-year follow-up, per-person costs were $2,740, $2,820, and $2,870.

**Table 4:** Model validation and 10-year outcomes for TB incidence and cost of 6EH and 36H compared to no IPT.

	Model Validation: 3 Year Outcomes			Model Projections: 10 Year Outcomes	
	Trial Data [9]	Model Projections			
Strategy	TB cases per 100 PY(95% CI)	TB cases per100 PY	Discounted mean per person cost, 2009 USD	TB cases per 100 PY	Discounted mean per person cost, 2009 USD
**No IPT**	n/a	4.54	680	4.47	2,740
**6EH**	2.44 (1.42–3.46)	2.23	720	3.62	2,820
**36H**	1.55 (0.73–2.36)	1.74	770	3.44	2,870

**TB**: Tuberculosis; **PY**: Person-Years; **USD**: US Dollars; **IPT**: Isoniazid-based TB preventive therapy; **6EH**: Six-month regimen of isoniazid plus ethambutol; **36H**: Three-year regimen of isoniazid.

In the absence of IPT, the projected discounted life expectancy was 136.1 months, with a discounted mean per person lifetime cost of $5,630 (Table 5). 6EH increased the discounted life expectancy to 136.9 months at an additional cost of $101 per person, resulting in an incremental cost-effectiveness ratio of $1,490/YLS. The life expectancy with 36H was 137.1 months at an additional cost of $55 compared to 6EH, resulting in an incremental cost-effectiveness ratio of $3,120/YLS.

**Table 5:** Incremental cost-effectiveness of TB preventive therapy for HIV-infected individuals in India.

Strategy	Discounted mean per person lifetime cost, 2009 USD	Discounted mean person life expectancy, months (undiscounted)	Incremental Cost-effectiveness ratio, $/YLS
**No IPT**	5,630	136.1 (184.5)	–
**6EH**	5,730	136.9 (185.6)	1,490
**36H**	5,780	137.1 (185.8)	3,120

**TB**: Tuberculosis; **PY**: Person-Years; **USD**: US Dollars; **IPT**: Isoniazid-based TB preventive therapy; **6EH**: Six-month regimen of isoniazid plus ethambutol; **36H**: Three-year regimen of isoniazid; YLS: Year of life saved.

### Sensitivity Analyses: 6EH Compared to No IPT

**Figure 1:**
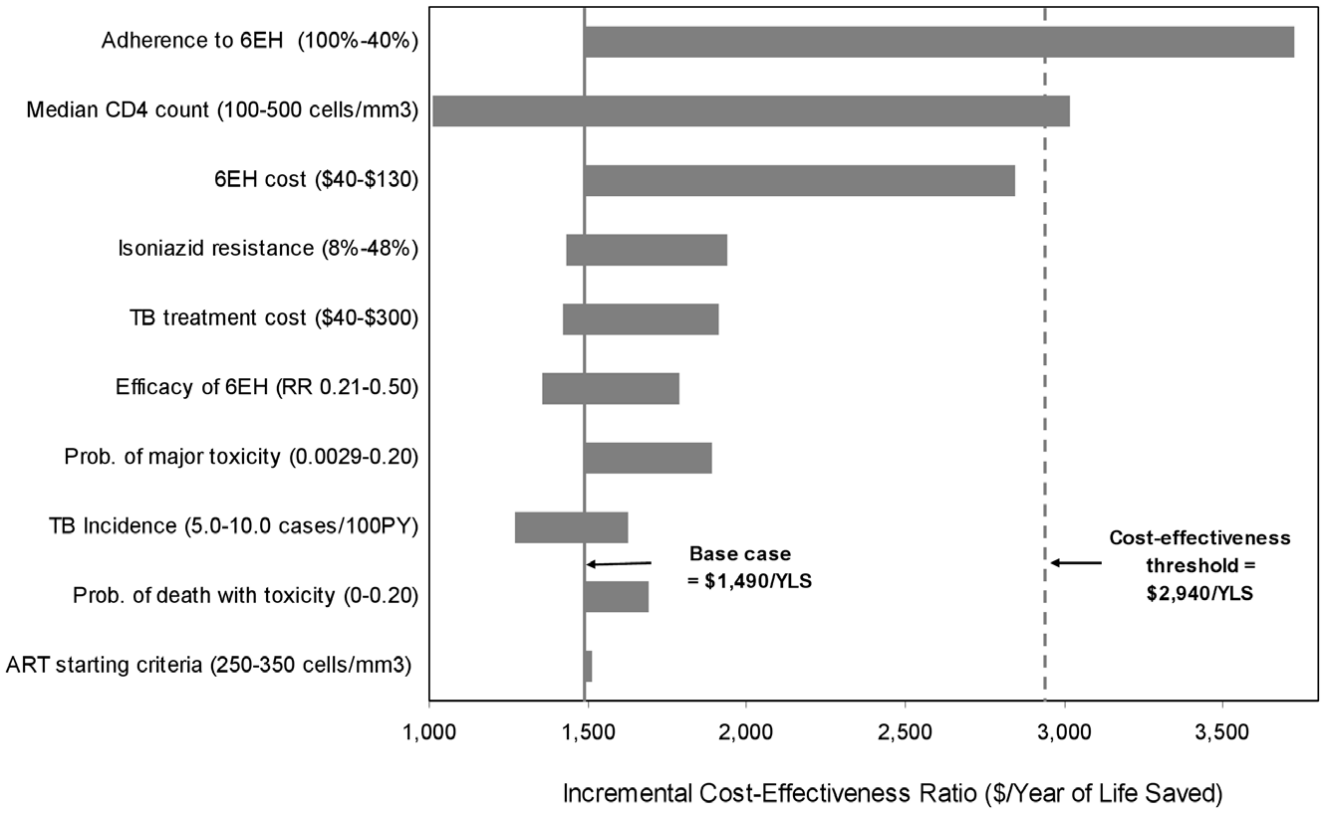
Tornado diagram of one-way sensitivity analyses comparing the 6EH regimen to no IPT . Selected model parameters are listed on the vertical axis, with the range examined in sensitivity analyses listed in parentheses. The length of the horizontal bar demonstrates the impact of changes in the parameter values on the incremental cost-effectiveness ratios for the 6EH regimen compared to no IPT. The solid vertical line indicates the incremental cost-effectiveness ratio estimate ($1,490/YLS) of the base case, and the dashed vertical line indicates the suggested cost-effectiveness threshold ratio of $2,940/YLS (3x GDP India). Values in white in the center of the bars indicate the threshold value of each parameter at which the cost-effectiveness ratio for 6 months of IPT compared to no IPT is equal to $2,940/YLS. For example, Adherence to the 6EH regimen was varied from 40%–100%. The incremental cost-effectiveness ratio of 6EH compared to no IPT was less than or equal to $2,940/YLS (i.e. cost-effective by international standards) when percent cohort completion was as low as 55%. **6EH**: Six-month regimen of isoniazid plus ethambutol, **IPT**: Isoniazid-based Preventive Therapy, **YLS**: Year of Life Saved, **GDP**: Gross Domestic Product.

In one-way sensitivity analysis, 6EH remained cost-effective compared to no IPT (with an incremental cost-effectiveness ratio below the threshold of $2,940/YLS, or 3x GDP *per capita* of India) when the mean CD4 count was <490 cells/mm^3^ at baseline, when at least 55% of the total cohort completed the 6EH regimen, and when the cost of the 6EH regimen was increased to $130 reflecting intensified case-finding with three AFB smears and cultures plus chest radiograph at initial screening, as well as two smears and cultures plus chest radiograph every six months thereafter (Figure 1). When the efficacy of 6EH was varied between the bounds of the 95% confidence interval of the point estimates observed in the trial, the incremental cost-effectiveness ratios ranged from $1,360/YLS to $1,790/YLS. The incremental cost-effectiveness ratios remained stable with wide variations in other parameters, including increasing the probability of major toxicity to 20%, increasing the probability of death from major toxicity to 20%, increasing the cost of active TB by 500%, tripling the prevalence of isoniazid-resistant TB to 48% (with prevalence of MDR-TB increased to 18%), ranging the overall incidence of TB from 5 to 10 cases/100 PY, and expanding the CD4 ART initiation to ≤350_,_ cells/mm^3^. Results were stable with wide variation in rates of CD4 decline, CD4-specific mortality, and ART efficacy.

**Figure 2:**
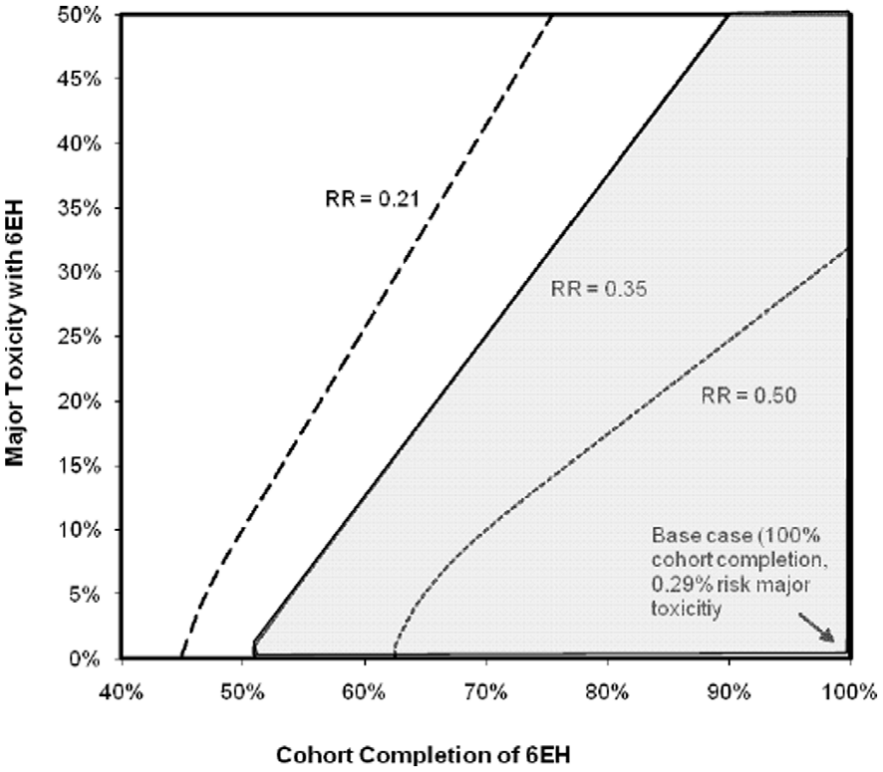
Multi-way sensitivity analysis of 6EH major toxicity, regimen completion, and efficacy . Major toxicity associated with 6EH, percent cohort completion of the regimen, and efficacy of the regimen are varied simultaneously. The solid line represents the point estimate of efficacy for the 6EH regimen as observed in the trial, i.e. a rate ratio of tuberculosis incidence of 0.35 compared to no IPT, varied over a range of percent cohort completion of the regimen and probability of major toxicity. The shaded area to the right of the line represents all values at which the 6EH regimen is cost-effective, based on 3x the GDP *per capita* of India. The hatched line represents the leftwards shift in the boundary of cost-effectiveness when the rate ratio of TB incidence with 6EH is 0.21 compared to no IPT, as calculated from the lower bound of the 95% confidence interval of the trial. The dotted line represents the rightwards shift in the boundary of cost-effectiveness when the relative risk of TB with 6EH is 0.50 compared to no IPT, as calculated from the upper bound of the 95% confidence interval of the trial. The diamond in the lower right corner represents the base case, trial-based scenario, where percent cohort completion is 100% and the probability of major toxicity is 0.0029. **6EH:** Six-month regimen of isoniazid plus ethambutol, **IPT**: Isoniazid-based Preventive Therapy, **GDP**: Gross Domestic Product.

In a multi-way sensitivity analysis, the incremental cost-effectiveness of 6EH remained ≤$2,940/YLS under the most optimal assumption of efficacy (RR = 0.21, representing the lower bound of the 95% confidence interval for the point estimate of efficacy in the trial) when the probability of toxicity was as high as 20% and at least 56% of the cohort completed the regimen (Figure 2). Using the least optimal assumption for efficacy (RR = 0.50, representing the upper bound of the 95% confidence interval), the regimen remained cost-effective if the probability of major toxicity was less than 10% and at least 69% of the cohort completed the regimen.

### Sensitivity Analyses: 36H Compared to 6EH

**Figure 3:**
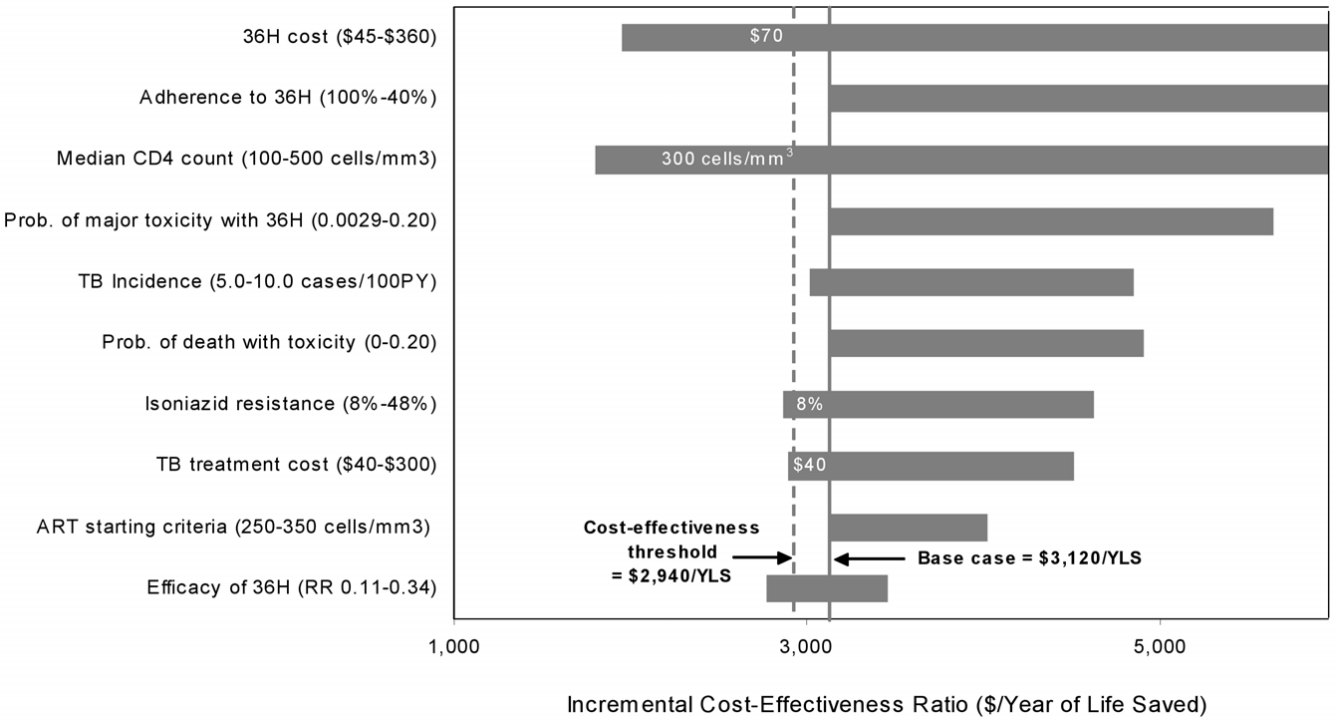
Tornado diagram of one-way sensitivity analyses comparing the 36H regimen to the 6EH regimen . Selected model parameters are listed on the vertical axis, with the range examined in sensitivity analyses listed in parentheses. The horizontal axis demonstrates the impact of changes in the parameter values on the incremental cost-effectiveness ratios for the 36H regimen compared to 6EH regimen. The solid vertical line indicates the incremental cost-effectiveness ratio estimate ($3,120/YLS) of the base case, and the dashed vertical line indicates the suggested cost-effectiveness threshold ratio of $2,940/YLS (3x GDP India). Values in white in the center of the bars indicate the threshold value of each parameter at which the cost-effectiveness ratio for 36H compared to 6EH is equal to $2,940/YLS. For example, a total cost of $90 for the 36H regimen was assumed in the base case. The incremental cost-effectiveness ratio of 36H compared to 6EH was less than or equal to $2,940/YLS (i.e. cost-effective by international standards) when the cost of 36H was less than $70. **36H:** Thirty-six-month regime of isoniazid, **6EH**: Six-month regimen of isoniazid plus ethambutol, **YLS**: Year of Life Saved, **GDP**: Gross Domestic Product.

Assuming a significant difference between the efficacy of 36H and 6EH, the impact of the mean CD4 count, regimen cost, and percent cohort completion had similar impact on cost-effectiveness when parameters were individually varied (Figure 3). The 36H regimen was cost-effective compared to 6EH when the cost was reduced to $70, the median CD4 was decreased to 300 cells/mm^3^, and when the prevalence of INH-resistant TB was decreased to 8%. A multi-way sensitivity analysis was performed to examine a best case scenario in which the point estimate for the 36H regimen was significant, the cost of the regimens was reduced to $20 by excluding clinic visits and liver function test costs (assuming these would be included in routine HIV costs when HIV and TB care were co-localized) and ART was initiated at a CD4 count ≤350 cells/mm^3^. In this scenario, the 36H regimen was more cost-effective than the 6H regimen, with an incremental cost-effectiveness ratio, compared to no IPT, of $1,130/YLS.

### Expanded IPT Strategies

When additional IPT strategies were considered, 6H resulted in a projected life expectancy of 136.9, with a mean, discounted per person lifetime cost of $5,700, whereas 3RH increased projected life expectancy by 0.03 months at a per person lifetime cost of $5,710 (Table 6). The 6EH strategy was eliminated by extended dominance (less cost-effective than other more effective strategies) due to the increased per person lifetime cost without associated mortality benefit relative the 3RH regimen. When the efficacy of the 6EH regimen was increased to represent the lower bound of the 95% confidence interval for this regimen (TB incidence rate ratio = 0.21), as observed in the trial setting, the strategy was no longer dominated, and had an incremental cost-effectiveness ratio of $2,440/YLS (data not shown).

**Table 6:** Incremental cost-effectiveness of additional strategies for TB preventive therapy.

Strategy	Discounted mean per person lifetimecost, 2009 USD	Discounted mean person life expectancy, months	Incremental cost-effectivenessratio, $/YLS*
**No IPT**	5,630	136.1	–
**6H**	5,700	136.9	1,140
**3RH**	5,710	136.9	1,610
**6EH**	5,730	136.9	Dominated^a^
**36H**	5,780	137.1	4,290
**3RPTH**	5,860	136.9	**Dominated** b

**TB**: Tuberculosis; **USD**: US Dollars; **YLS**: Years of life saved: **IPT**: Isoniazid-based preventive therapy; **6H**: Six-month regimen of isoniazid; **3RH**: Three-month regimen of isoniazid plus rifampin; **6EH**: Six-month regimen of isoniazid plus ethambutol; **36H**: Three-year regimen of isoniazid; **3RPTH**: Three-month regimen of isoniazid plus rifapentine.

* The incremental cost-effectiveness ratios may not exactly match the ratios of lifetime cost and life expectancy reported in the table due to rounding.

a Weakly dominated (more expensive but confers less clinical benefit than some combination of other strategies) [12].

b Strongly dominated (more expensive but confers less clinical benefit than some other strategy) [12].

When compared to the 3RH regimen, the incremental cost-effectiveness of 36H was $4,290/YLS. Compared to 36H, per person lifetime cost increased by $80 for the 3RPTH regimen and was associated with decreased life expectancy.

## Discussion

Our analysis demonstrated that a six-month course of isoniazid-based preventive therapy for HIV-infected individuals decreased TB incidence, increased overall life expectancy and was cost-effective in India. These findings were robust across wide variations in parameters including adherence, prevalence of drug-resistant TB, drug toxicity, and costs. The thirty-six month regimen may be considered more cost-effective than the six month regimen in the best case scenario when efficacy was significantly greater than the six-month regimen, only marginal costs of IPT medications were considered in the case of co-localized TB/HIV care, and ART was initiated earlier.

Several recent trials have examined various IPT regimens for HIV-infected individuals in resource-limited settings, including six months versus continuous isoniazid in Botswana, and short courses of rifampin and rifapentine containing regimens in South Africa [20], [21], [44]. Compared to 6H and 3RH, the 6EH regimen offered comparable efficacy at increased cost. When the efficacy of 6EH was varied within the 95% confidence interval of trial observations, the regimen became cost-effective, suggesting marginal differences in the clinical and economic impact of these regimens. 3RPTH, while conferring similar efficacy, was considerably more costly, and was therefore not found to be cost-effective in comparison to other regimens. While improved adherence and toxicity associated with four months of rifampin alone has been demonstrated in published studies, this was not evaluated in the current analysis as efficacy for the regimen has not yet been established in large scale clinical trials [45]. Additionally, concern for the theoretical risk of rifampin resistance in the setting of monotherapy has been raised [46].

The study differs from prior cost-effectiveness analyses of IPT in HIV-infected patients given greater availability of antiretroviral therapy [43], [47], [48]. In an analysis by Bell et. al. in the pre-ART era, six months isoniazid extended life expectancy from 7.79 to 8.37 years, and increased cost per person from $25.30 to $38.31 with an incremental cost-effectiveness ratio of $114/QALY [47]. Other pre-ART studies in Zambia and Cambodia found IPT to be cost-saving [43], [48]. In this analysis ART increased CD4 counts, thereby reducing the risk of TB and related mortality independent of IPT. This attenuation of the absolute IPT efficacy, coupled with the cost of chronic HIV care explains the findings of longer life expectancy, increased lifetime cost, and a higher cost-effectiveness ratio. The six-month regimen remained cost-effective when the CD4 threshold for ART initiation was increased to ≤350 cells/mm^3^ suggesting that IPT remained a good value for money as countries expand access to ART, and could further support IPT given recent studies examining the complementary effects of IPT and ART [17].

Critics of IPT cite the potential increased risk of hepatotoxicity in the setting of expanded ART [49]. In a recent clinical trial in Botswana of IPT in HIV-infected patients, 1.1% of subjects developed severe hepatitis, of whom 5% died [50]. In sensitivity analysis, six months of IPT remained cost-effective well within this range of toxicity.

Risk of undiagnosed TB at time of IPT initiation, poor adherence, and drug-resistance are other cited barriers to the success of IPT [49], [51], [52]. In the current analysis, six months of IPT remained cost-effective when the cost of intensified case-finding, defined by TB smear and culture times three plus chest radiograph at IPT initiation, and smear and culture times two plus chest radiograph every six months thereafter were included. In regards to adherence, six months of IPT remained cost-effective even if 45% of the population did not complete the regimen. This is consistent with a cohort study performed in South Africa where adherence as low as 59% conferred a 27% reduction in the incidence of TB [53]. Shorter courses of rifamycin-containing IPT may abrogate this issue further still given higher rates of adherence in these regimens [20], [44]. When the prevalence of drug-resistant TB was increased to 48%, six months of IPT remained cost-effective. In most developing countries, including India, the prevalence of primary isoniazid resistance varies from 5 to 27% [54].

These findings should be interpreted within the context of certain limitations. Efficacy calculations were derived comparing trial data to natural history data for TB incidence as there was no placebo arm in recent trials. This is a benefit of model-based studies which can compare strategies that would otherwise be unethical to evaluate in a trial. The difference in efficacy of 36H compared to 6EH did not reach statistical significance in the trial. Projections of the 36H regimen suggest that it may be cost-effective as compared to the 6EH regimen in the best case scenario. Several data sources, including the efficacy of additional IPT regimens, mortality associated with AIDS and OI from Côte d’Ivoire were used to populate the model when Indian data were not available. These assumptions were extensively tested in sensitivity analysis. Previous studies have found greater IPT efficacy in patients with positive tuberculin skin testing [38]. This strategy was not examined as TB incidence was not significantly different when stratified by skin test <5 mm and ≥ 5 mm in either arms of the National Institute for Research in Tuberculosis trial [9]. Finally, secondary TB transmission was not modeled, likely underestimating the benefits of IPT given prevention of TB transmission at no additional cost.

The magnitude of TB-associated morbidity and mortality in HIV-infected patients has prompted the government of India to enhance interventions addressing both diseases [7]. The safety and efficacy of isoniazid-based preventive therapy has been demonstrated in this setting [9]. This analysis shows that a three-month course of isoniazid plus rifampin and a six-month of isoniazid alone both decrease TB incidence and are similarly cost-effective by WHO criteria. Inclusion of isoniazid-based therapy as part of HIV care represents a critical and cost-effective opportunity to enhance TB control and improve outcomes for HIV-infected patients.
